# When fecundity does not equal fitness: evidence of an offspring quantity versus quality trade-off in pre-industrial humans

**DOI:** 10.1098/rspb.2007.1000

**Published:** 2008-01-23

**Authors:** Duncan O.S Gillespie, Andrew F Russell, Virpi Lummaa

**Affiliations:** Department of Animal and Plant Sciences, University of SheffieldSheffield S10 2TN, UK

**Keywords:** life-history evolution, maternal fitness, recruitment, reproductive success, resource acquisition, socio-economic status

## Abstract

Maternal fitness should be maximized by the optimal division of reproductive investment between offspring number and offspring quality. While evidence for this is abundant in many taxa, there have been fewer tests in mammals, and in particular, humans. We used a dataset of humans spanning three generations from pre-industrial Finland to test how increases in maternal fecundity affect offspring quality and maternal fitness in contrasting socio-economic conditions. For ‘resource-poor’ landless families, but not ‘resource-rich’ landowning families, maternal fitness returns diminished with increased maternal fecundity. This was because the average offspring contribution to maternal fitness declined with increased maternal fecundity for landless but not landowning families. This decline was due to reduced offspring recruitment with increased maternal fecundity. However, in landowning families, recruited offspring fecundity increased with increased maternal fecundity. This suggests that despite decreased offspring recruitment, maternal fitness is not reduced in favourable socio-economic conditions due to an increase in subsequent offspring fecundity. These results provide evidence consistent with an offspring quantity–quality trade-off in the lifetime reproduction of humans from poor socio-economic conditions. The results also highlight the importance of measuring offspring quality across their whole lifespan to estimate reliably the fitness consequences of increased maternal fecundity.

## 1. Introduction

The life-history trade-off between the number of offspring produced and their quality is a fundamental idea in evolutionary biology (Lack 1947; Smith & Fretwell 1974; Stearns 1992). It assumes that resources are limited and is based on three principles: (i) as investment in offspring number is increased, investment per offspring is decreased, (ii) increased investment in each offspring enhances offspring reproductive success, and (iii) maternal fitness (i.e. a mother's contribution to population growth) is determined by the number of offspring recruited into the breeding population (i.e. those that breed) and their subsequent lifetime reproductive success (LRS; Roff 2002). The original formulation of the offspring quantity versus quality hypothesis by Lack (1947) used these principles to suggest that maternal fecundity in natural populations represents an evolved balance between offspring number and quality, which maximizes maternal fitness. This led to the general expectation that maternal genotypes reflect a process of natural selection that has favoured an optimal division of resources between offspring quantity and quality (Falconer 1989; Stearns 1992). However, few studies have investigated the effects of increased maternal fecundity on overall offspring quality (i.e. both offspring recruitment and their subsequent reproductive success) in wild populations.

Some of the best evidence for a trade-off between offspring quantity and quality in the wild has come from experimental studies of bird populations such as collared flycatchers (*Ficedula albicollis*: Gustafsson & Sutherland 1988) and kestrels (*Falco tinnunculus*: Dijkstra *et al.* 1990). These studies showed that offspring from enlarged clutches have a lower recruitment probability and lower clutch sizes in their first year of breeding than offspring from natural clutch sizes. A similar study of the bank vole (*Clethrionomys glareolus*) showed that offspring from enlarged litters can have a lower weaning mass and recruitment probability, but appear not to have reduced litter sizes in their first year of breeding (Koskela 1998). However, few studies in the wild have been able to follow offspring throughout their lives and to record their LRS, or have been able to link variation in LRS with measures of resource availability to breeding females. The need for such information was highlighted by a laboratory study of the parasitoid wasp, *Goniozus nephantidis* (Hardy *et al.* 1992). This showed that enlarged clutches had little effect on offspring survival, but did reduce offspring size and subsequent fecundity due to increased resource competition within the clutch. Thus, without reliable information on the traits influencing offspring LRS, there is a risk of misrepresenting the costs to offspring and the fitness benefits to mothers of increased brood size. That the most common clutch size in wild bird populations is often below the calculated optimum for maximal maternal fitness suggests that this problem could be real (e.g. Dijkstra *et al.* 1990; but see Godfray *et al.* (1991) for a discussion of potential deviations from the optimal clutch size).

In humans, a trade-off is expected between maternal lifetime fecundity and offspring quality due to the need to divide limited resources between several simultaneously dependent offspring (Kaplan 1996; Hill & Kaplan 1999; Gurven & Walker 2006). Human studies offer a way around the problem of tracking individuals throughout life because the LRS of all offspring may be extracted from census records (e.g. Lahdenperä *et al.* 2004). Importantly, there is also evidence suggesting that humans are sensitive to a trade-off between offspring quantity and quality. For example, the production of twins has been shown to increase maternal fitness only where resources are abundant and relatively constant (Lummaa *et al.* 1998). In addition, intermediate inter-birth intervals and offspring numbers have been shown to correspond with maximal offspring survivorship (Blurton Jones 1997; Strassmann & Gillespie 2002; Mace 2007). Finally, increased birth rates can also increase malnutrition among offspring (Gibson & Mace 2006).

However, some studies of contemporary hunter-gatherer populations have not shown the expected trade-off, but instead observed that offspring quality increases rather than decreases with increasing maternal fecundity or birth rate (Pennington & Harpending 1988; Hill & Hurtado 1996). These observations might be due to the particular measures of offspring used in the studies or social or ecological situations within these communities. For example, other studies have indicated that the maternal fitness derived from different levels of maternal fecundity may be dependent on individual wealth or social status (Borgerhoff Mulder 2000; Hagen *et al.* 2006). Thus, individuals with easy access to abundant resources might experience a relaxation of the reproductive constraints imposed by nutrition and workload, and this could lead to observed positive correlations between traits that are expected to trade-off (van Noordwijk & de Jong 1986; Mace 1996). These conflicting results have led to unresolved questions regarding the existence of an overall quantity versus quality trade-off in human lifetime reproduction (Hill & Hurtado 1996; Blurton Jones 1997; Hill & Kaplan 1999), and a major reason for this is the difficulty of linking maternal fecundity directly with subsequent offspring fecundity and maternal fitness.

The aim of our study was to investigate the presence of a trade-off between the number of offspring (i.e. maternal fecundity) versus offspring recruitment and subsequent offspring fecundity under contrasting socio-economic conditions. We used a three-generation, individual-based dataset of pre-industrial Finns from the eighteenth and nineteenth centuries. These demographic data have at least three benefits for addressing our aim. First, we have reliable information about the total number of offspring and grand-offspring of each woman in our dataset. This is because it was collated from church registers maintained by local clergymen who were obliged by law to submit to the state accurate records of the survival and reproductive history of all individuals in their parish area (Luther 1993). Migration rates were low and, in most cases, the parish migration registers allow the LRS of dispersers to be determined. Second, the study period coincides with periods of natural fertility and mortality and ends before healthcare and more liberal economics improved standards of living in Finland (Soininen 1974). Third, the data include the socio-economic status of each woman, assigned according to her husband's occupation. These socio-economic data can be taken to represent differences in resource availability in terms of nutrition, wealth and workload between individuals, which have already been shown to influence long-term fitness in our study population (Pettay *et al.* 2007) as well as other pre-industrial populations (e.g. Voland 1990).

We first investigate how both maternal fecundity and the proportion of offspring recruited (i.e. survived to breed) combine to predict total grand-offspring number in contrasting socio-economic conditions. We use total grand-offspring number as our measure of maternal fitness. This enables us to link offspring number to maternal fitness through the effects of offspring recruitment (survival to breed) and the subsequent fecundity of recruited offspring. Second, for each socio-economic condition, we focus on how maternal fecundity specifically predicts three measures of offspring quality. Our three measures of offspring quality are as follows. (i) The average number of grand-offspring that each birth contributed to maternal fitness, as an overall measure of offspring quality. This combines both offspring recruitment and subsequent offspring fecundity, with higher recruitment and subsequent fecundity indicating higher quality. (ii) The proportion of offspring recruited. (iii) Subsequent offspring fecundity. In keeping with Lack's hypothesis, we predict that in poor socio-economic conditions, maternal fecundity beyond the population mean will lead to diminishing returns in maternal fitness due to reductions in offspring quality. Conversely, in favourable socio-economic conditions, maternal fecundity beyond the population mean should continue to give proportional returns in maternal fitness due to a relaxation of constraints on offspring quality.

## 2. Material and methods

Our data contain three generations of full reproductive history and survival details collected by genealogists for women sampled from four geographically isolated parishes in Finland: Ikaalinen (61°45′ N, 23° E); Hiittinen (60° N, 22°30′ E); Kustavi (60°30′ N, 21°30′ E); and Rymättyla (60°15′ N, 22° E). From these parish church registers, we obtained a sample of 446 women who gave birth to at least one offspring in their lifetime. Of these, only those whose complete lifetime fecundity could be reliably determined were included in the analysis (resulting sample sizes are shown in table 1). All presented results are from this full dataset of 437 women, who were born between 1709 and 1815. These women had a total of 2888 offspring born from 1741 to 1858, and 6470 grand-offspring born from 1766 to 1901. Our study period thus ended before healthcare and more liberal economics began to improve standards of living in Finland (Soininen 1974). In addition, in most parts of the country during our study period (1709–1901), frosty nights during early summer and rain at harvest time in the autumn often led to unpredictable crop failures and subsequent famines, and even during average harvest years, 5–10% of people consumed emergency foods (Jutikkala *et al.* 1980). During famines, the most common causes of death were infectious diseases such as tuberculosis, cholera, scarlet fever and smallpox, which readily spread among the malnourished people (Turpeinen 1978). Indeed, infection is likely to have been the primary cause of infant mortality, particularly among poorer families (Moring 1998).

Socio-economic status was initially assigned with advice from historians, on the basis of occupations that had similar access to wealth and food (see Pettay *et al.* 2007). As women rarely had a profession, their socio-economic status was the same as their husband's at the time the offspring were born. We simplified the socio-economic status classification into two groups, hereafter referred to as landowners and landless, according to those owning land versus those either renting or having no access to land at all. Although there is likely to be high levels of variation within these groups, this allowed us to observe the major effects of differences in socio-economic status. Importantly, landownership in pre-industrial populations has been shown to have effects on both fecundity (Easterlin 1976) and other life-history traits such as offspring survival and marriage probability (Voland & Dunbar 1995). During the study era, inheritance usually favoured the eldest son. The mating system was monogamous to an unusually high degree, with almost all reproductive individuals married and divorce forbidden (Moring 1993).

We carried out all statistical analysis in the R environment (v. 2.4.1; R Development Core Team 2006). All *p*-values are two-tailed. It is worth noting that a 95% CI which includes zero is equivalent to a *p*-value greater than 0.05. One potential problem is that our results might be modified by women who died before they reached menopause, if such women produced few, low-quality children owing to dying young. We therefore repeated each analysis outlined below without women who died before age 50, which was the latest age of reproduction in the dataset (reduced dataset size of 363 women). Similarly, women whose husbands died before those women reached menopause might also modify the results. This is because husband's death could reduce both offspring number and offspring quality due to decreased family income. Therefore, we also repeated each analysis using only women who both survived to age 50 and whose first husbands were alive when they reached this age (reduced dataset size of 240 women). However, we found that the results using these reduced datasets were qualitatively similar to those from the full dataset of 437 women.

Preliminary to the main analyses outlined in §2*a*,*b*, we initially described the differences in the numbers of offspring, recruited offspring and grand-offspring between the landowning and landless socio-economic groups. To do this, we used three separate linear models with Box–Cox power-transformed response variables (table 1). The Box–Cox powers for offspring (0.68), recruited offspring (0.59) and grand-offspring (0.43) were identified using the function *boxcox* in the R package *MASS*. These descriptive analyses were used to highlight the general effects of socio-economic differences before contrasting the relationship between maternal fecundity and offspring quality or maternal fitness between the socio-economic groups.

### (a) Maternal fecundity, offspring recruitment and grand-offspring number

We first investigated the influence of socio-economic conditions on the way maternal fecundity and the proportion of offspring recruited combine to predict total grand-offspring number. This was done using a generalized linear mixed model (GLMM) with a Poisson error structure and a log link function that was fitted using the function *lmer* in the R package *lme4* (Bates & Sarkar 2007). To the model, we added random intercepts for parish (four levels) and maternal birth cohort (10-year periods) to correct for geographical and temporal variation in fecundity and recruitment. However, the addition of maternal birth cohort to the model explained only a very small amount of additional variation. The model was fitted to the full dataset of 437 women using a Laplace approximation to maximum likelihood. Despite the quality of the church registers, it was occasionally not possible to determine reliably the total number of grand-offspring because some offspring (9%) were not followed for their entire life history. We accounted for this by including a vector of weights in the model, according to the proportion of a mother's offspring with complete follow-up.

Initial data inspection indicated that both maternal fecundity and the proportion of offspring recruited had curvilinear (quadratic) relationships with grand-offspring number (figure 1). Therefore, we also added quadratic terms for each of these explanatory variables to the model. To visualize the three-dimensional surface created by the quadratic relationships of these two explanatory variables with grand-offspring number, we used a second-order response surface model. This is a standard method for describing three-dimensional surfaces where there might be maxima, minima or ridges in the response variable (in this case, grand-offspring number subject to a log link function). The equation for this model has the form *y*=*x*_1_+*x*_1_^2^+*x*_2_+*x*_2_^2^+*x*_1_*x*_2_ (Draper & John 1988). To investigate the influence of socio-economic status, we compared a model, where *x*_1_ is the maternal fecundity and *x*_2_ the proportion of offspring recruited, with an expanded model that incorporated socio-economic status as a two-level factor (landowners versus landless) and its interaction with each term in the smaller model (Draper & John 1988). Comparison between these two models was made based on standard likelihood-ratio tests as well as the difference in Akaike's information criterion (AIC; table 2).

To allow the assessment of uncertainty in the estimates of individual parameters in the model that included socio-economic status, we reduced this model to its minimal adequate model (MAM). This was done by single-term deletion, starting with the highest-order interactions. We discarded terms if their presence did not improve the explanatory power of the model. This was tested using both model AIC and standard likelihood-ratio tests (table 2). The use of whole-model deviance in this way is equivalent to assessing the deviance explained by individual terms in the model, e.g. using *F*-ratios in a traditional ANOVA. However, *F*-ratios are currently unobtainable for the type of model used here. The predicted values from the resulting MAM were then interpolated and used to generate contour plots showing the relationships of maternal fecundity and offspring recruitment with grand-offspring number in each socio-economic group.

Uncertainty in the MAM parameter estimates was assessed primarily using 95% confidence limits based on the underlying Poisson distribution of the model residuals. To do this, we first generated a Markov Chain Monte Carlo (MCMC) sample from the posterior distribution of each parameter estimate. This used the function *mcmcsamp* in the R package *lme4* (Bates & Sarkar 2007). Second, we computed the Bayesian highest posterior density (HPD) 95% CIs of the MCMC sample for each parameter estimate using the function *HPDinterval* in the R package *coda* (Plummer *et al.* 2006). Although this method does not generate *p*-values, it is currently the most reliable way to assess the uncertainty in the parameter estimates for this type of GLMM (Baayen *et al.* submitted). However, despite the potential bias, for ease of interpretation, we have also provided *p*-values based on the normal (*z*) distribution.

### (b) Offspring quantity versus quality

To investigate the relationship between maternal fecundity and offspring quality (rather than overall maternal fitness) in each socio-economic group, we used three different measures of offspring quality. (i) As a measure of overall offspring quality, we used the average number of grand-offspring that each birth contributed to maternal fitness (i.e. grand-offspring per birth). This overall measure of offspring quality combined both the proportion of offspring recruited and their subsequent lifetime fecundity. Then, in order to understand the individual contributions of these two variables to offspring quality in each socio-economic group, we analysed the data separately for (ii) the proportion of offspring recruited and (iii) the subsequent fecundity of recruited offspring.

As offspring quality is ultimately a measure of the average contribution of each offspring to population growth, we needed to select an appropriate method to quantify offspring quality for each offspring number in each socio-economic group. One potential problem was that the high degree of temporal and spatial variation in our dataset (women were born over 106 years, 1709–1815, across Finland) leads to a large amount of variation in the average offspring quality of mothers with the same fecundity. This might bias any conclusions because increases in variation can decrease the long-term fitness associated with each maternal fecundity (Roff 2002). In order to account for any effect of this nature, we compared the geometric means of average offspring quality between women with each offspring number in each socio-economic group. The application of geometric means where there is random temporal or spatial variation can have a ‘variance discounting’ effect, which has been shown to increase the reliability of fitness estimates (Frank & Slatkin 1990). Geometric means have previously been used in theoretical investigations of offspring quantity versus quality because they can also assess the fitness outcomes of possible bet-hedging strategies where environmental conditions are variable (see Simons 2007). Finally, we compared the regression slopes of geometric mean offspring quality against maternal fecundity for the full dataset of 437 women between socio-economic groups, using an ANCOVA design in three separate linear models. This allowed the comparison of the effects of increased maternal fecundity on offspring quality in different socio-economic conditions. The response variables in these linear models met the assumptions of normality.

## 3. Results

The initial descriptive analyses of the differences between socio-economic groups showed that women from landowning families had higher numbers of offspring (i.e. maternal fecundity), more recruited offspring and more grand-offspring (i.e. maternal fitness) than women from landless families (table 1).

### (a) Maternal fecundity, offspring recruitment and grand-offspring number

Socio-economic status had a significant influence on the way maternal fecundity and the proportion of offspring recruited combined to predict grand-offspring number (table 2). Improved socio-economic conditions increased the number of grand-offspring gained from increased maternal fecundity (table 3). As the model in table 3 accounts for the effects of offspring recruitment, this suggests that the subsequent fecundity of offspring in landowning families remained higher than in landless families as maternal fecundity increased. This can be seen in figure 2*a*,*b* by following the horizontal dashed line, which indicates the population mean of the proportion of offspring recruited. For landowning but not landless families, the number of grand-offspring continues to increase with increasing maternal fecundity. Progressive exclusion of women with the highest fecundity confirmed that the relationship between maternal fecundity and grand-offspring number was not significantly curvilinear below seven births for landless families. Notably, this is remarkably close to the population mean maternal fecundity (6.61, s.e.±0.13). This suggests that for landless families, grand-offspring returns begin to diminish at maternal fecundities beyond the population mean. These results were qualitatively the same when we excluded from the analysis women who died before age 50, or those whose husbands died before the women reached age 50.

After accounting for the effects of maternal fecundity, grand-offspring number showed a significantly curvilinear relationship with the proportion of offspring recruited, which did not differ significantly between socio-economic groups (table 2). The presence of the same curvilinear relationship in both socio-economic groups suggests that increased offspring recruitment leads to increased competition among recruited offspring. This might lead to the observed diminished grand-offspring returns due to the association between socio-economic conditions and fecundity (see §4). This is supported by the finding that the relationship between offspring recruitment and grand-offspring number (figure 1*b*) does not appear significantly curvilinear below the population mean of the proportion of offspring recruited (0.47, s.e.±0.01). In addition, it is worth noting that the linear relationships of maternal fecundity and offspring recruitment with grand-offspring number were significantly steeper for women from landless families (table 3). This is likely to be because the highest numbers of grand-offspring in the landless socio-economic group are associated with higher levels of offspring recruitment at lower maternal fecundities than in the landowning group (figure 2).

### (b) Offspring quantity versus quality

To investigate the relationship between maternal fecundity and offspring quality in each socio-economic group, we used three different measures of offspring quality (figure 3; table 4). (i) As a measure of overall offspring quality, we used the average number of grand-offspring that each birth contributed to maternal fitness (i.e. grand-offspring per birth). Then, we analysed the data separately for (ii) the proportion of offspring recruited and (iii) the subsequent fecundity of recruited offspring.

First, women from landowning families with high maternal fecundity were able to maintain high overall offspring quality. However, this was not the case for women from landless families, which showed declines in overall offspring quality with increasing maternal fecundity (figure 3*a*,*b*; table 4). Notably, the regression line of overall offspring quality in landless families dropped below the population mean overall offspring quality at the population mean maternal fecundity. This indicates that for landless families, above-average maternal fecundity does indeed lead to below-average overall offspring quality. These results were qualitatively the same when we repeated the analysis without women who died, or whose husbands died before the women reached age 50.

Second, increased maternal fecundity caused the proportion of offspring recruited to decrease and this was not influenced by socio-economic grouping (figure 3*c*,*d*; table 4). However, as with previous studies, there is some ambiguity of cause and effect between maternal fecundity and early offspring survival (see §4), which is a major component of offspring recruitment. Even so, it is worth noting that the regression line of the proportion of offspring recruited dropped below the population mean of the proportion of offspring recruited at the population mean maternal fecundity. This suggests that producing more than the mean number of offspring leads to below-average recruitment of those offspring in both socio-economic groups.

Third, the subsequent offspring fecundity of women from landless families remained constant with increasing maternal fecundity. In contrast, the subsequent offspring fecundity of women from landowning families increased with increasing maternal fecundity (figure 3*e*,*f*; table 4). Thus, for women from landowning families, high maternal fecundity also led to subsequently high offspring fecundity, despite the correspondingly low offspring recruitment described above. The high subsequent offspring fecundity in landowning families appeared to ‘compensate’ for the potential decrease in overall offspring quality caused by decreased offspring recruitment. However, women from landless families appeared to exhibit a trade-off between the production of sufficient numbers of offspring and the achievement of sufficiently high recruitment of those offspring to maximize grand-offspring number.

## 4. Discussion

We took advantage of a multigenerational dataset of humans from pre-industrial Finland to test how maternal fecundity affects offspring quality and maternal fitness in landowning versus landless families. For women from landless families, subsequent offspring fecundity was constrained by increasing maternal fecundity, leading to lower grand-offspring returns compared with women from landowning families. We then specifically investigated two main aspects of offspring quality: the proportion of offspring recruited and the subsequent fecundity of recruited offspring. The proportion of offspring recruited decreased with increasing maternal fecundity and this was unaffected by socio-economic status. However, subsequent offspring fecundity increased with increasing maternal fecundity in landowning families, but remained constant with increasing maternal fecundity in landless families. Thus, maternal fitness showed diminishing returns with increasing maternal fecundity for women from landless but not from landowning families. These results are therefore consistent with Lack's (1947) hypothesis of a trade-off between offspring quantity and quality, and also with the expectation that such trade-offs should only be observed when resources are limited (van Noordwijk & de Jong 1986). In particular, the results suggest that the failure to observe an offspring quantity versus quality trade-off in some contemporary hunter-gatherer populations (e.g. Hill & Hurtado 1996) could be because the expected trade-off is masked by variation in socio-economic conditions not accounted for.

Few previous studies of offspring quantity versus quality in wild populations have been able to link maternal fecundity directly with subsequent offspring reproductive success and maternal fitness, which is fundamental to understanding the trade-off between offspring quantity and quality. Our results suggest that the use of more complete long-term data on offspring reproductive success could improve the consistency between the observed offspring numbers and the offspring numbers predicted to maximize maternal fitness. However, differences between observed and expected offspring numbers might arise for a number of adaptive reasons (see Godfray *et al.* 1991), rather than simply the inability to measure offspring fitness reliably. Nevertheless, our results suggest that the difference between the observed and expected offspring numbers could also be dependent on resource availability. For example, subsequent offspring fecundity increased with increasing maternal fecundity only for women from ‘resource-rich’ landowning families. This could be due to the association of high socio-economic status with lower age at first reproduction and increased fecundity among women in our study population (Pettay *et al.* 2007). Women from landowning families could thereby not only increase maternal fecundity, but also increase the socio-economic resources available to their offspring, and consequently increase subsequent offspring fecundity. Such a relaxation of socio-economic constraints on reproduction may be connected with the social transmission of reproductive behaviour leading to higher offspring fecundity (Anderton *et al.* 1987), and might also allow the realization of genetically inherited reproductive potential between mothers and daughters (Pettay *et al.* 2005).

Even so, in both socio-economic groups, we observed diminished grand-offspring returns beyond the population mean offspring recruitment. This indicates that regardless of socio-economic status, there is still competition among offspring for parental resources and investment. As such, our results are consistent with a study of an agro-pastoral community in Kenya, which showed lower grand-offspring returns for women with more than six or seven offspring surviving to 5 years of age, with this effect being strongest in the poorest families (Borgerhoff Mulder 2000). These results suggest that the trade-off between offspring quantity and quality is not necessarily concerned with the number of offspring born, but rather with the number of offspring that share parental resources over their entire juvenile period and perhaps beyond. Thus, the decrease in offspring recruitment with increasing maternal fecundity could be a result of compensation for early offspring mortality to optimize the number of offspring raised to independence. This idea that the degree of sibling competition for parental resources can be an important determinant of subsequent offspring reproductive success is consistent with research on the effects of early conditions in animals and humans (Lindström 1999; Lummaa & Clutton-Brock 2002). It is worth noting that whereas for many bird and mammal species early conditions are restricted to a short period before independence, the extended period of juvenile development in humans could prolong sensitivity to early conditions for many years (Gurven & Walker 2006). In addition, we have previously shown that high previous maternal reproductive investment (production of twins or more expensive sons) may decrease the mother's likelihood of re-breeding (Lummaa 2001) as well as the LRS of her subsequent offspring (Rickard *et al.* 2007). Finally, the costs to mothers of giving birth many times should not be forgotten, especially if these reduce maternal longevity (e.g. Helle *et al.* 2002), which in turn might reduce the amount of parental care available to surviving offspring (see Lahdenperä *et al.* 2004).

In conclusion, our study provides convincing evidence for the existence of a fundamental trade-off between offspring quantity and quality in human lifetime reproduction, which has no doubt shaped the evolution of human reproductive physiology and psychology. However, modern human societies that have undergone a demographic transition are somewhat enigmatic because women no longer give birth to the number of offspring that will maximize maternal fitness (Borgerhoff Mulder 1998). Even so, this need not contradict our conclusions when the modern labour market is considered (Kaplan 1996). For example, increasing delays and reductions in childbearing due to educational or career requirements appear consistent with a wish to acquire sufficient resources to give offspring ‘the best start in life’. This was illustrated by a study of the offspring quantity versus quality trade-off in New Mexican men between 1990 and 1993, which found no evidence for optimization based on fitness maximization but did find evidence for a trade-off between parental fecundity and subsequent offspring education and income (Kaplan *et al.* 1995). Thus, even though humans might still show adaptive reproductive behaviour, future investigators of this in terms of maternal fitness should be careful to take into account the particular socio-economic context of their study.

## Figures and Tables

**Figure 1:**
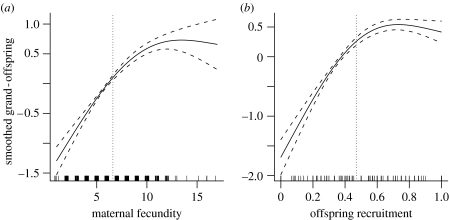
Grand-offspring number shows a significantly curvilinear (quadratic) relationship with both (*a*) maternal fecundity and (*b*) the proportion of offspring recruited (survived to breed). Solid lines represent non-parametric smoothing functions (on a standardized *y*-axis scale) of grand-offspring number, with dashed lines showing the standard errors. Vertical dotted lines show the population means of maternal fecundity (6.61) and the proportion of offspring recruited (0.47). Rug plots on the *x*-axis give an indication of the distribution of raw data points.

**Figure 2:**
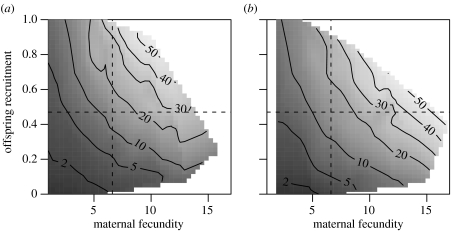
Increased maternal fecundity leads to lower grand-offspring returns for (*a*) women from landless families, compared with (*b*) women from landowning families. In addition, increases in the proportion of offspring recruited lead to lower grand-offspring returns in both socio-economic groups. Shown are the predicted values from the minimal adequate Poisson GLMM, with a log link function and random intercepts for each parish and 10-year maternal birth cohort. Contour lines represent different numbers of grand-offspring, with the number of grand-offspring shown on the contour line. The grey shaded area gives an indication of the range of raw data points. The population mean maternal fecundity (6.61) is shown by the vertical dashed lines and the proportion of offspring recruited (0.47) by the horizontal dashed lines.

**Figure 3:**
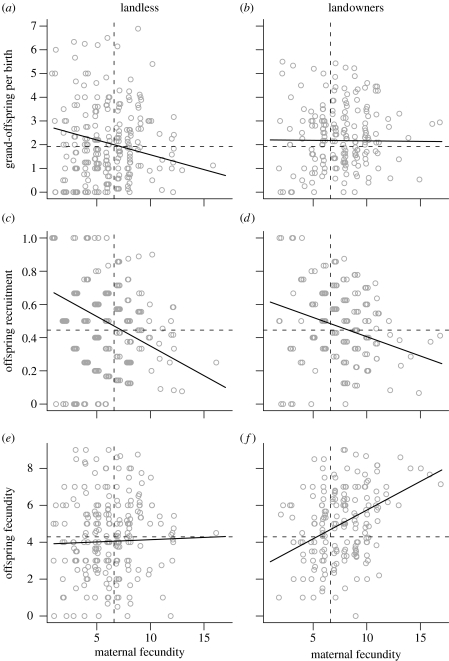
The linear model showed that (*a*) the number of grand-offspring per birth decreased with increasing maternal fecundity for women from landless families (slope estimate −0.12±0.044). (*b*) However, grand-offspring per birth remained constant with increasing maternal fecundity for women from landowning families (slope estimate 0.0043±0.040). Underlying these relationships were the following measures. (*c*,*d*) A decrease in the proportion of offspring recruited with increasing maternal fecundity in both socio-economic groups (slope estimate −0.028±0.0048). (*e*) Subsequent offspring fecundity remained constant with increasing maternal fecundity for women from landless families (slope estimate 0.024±0.044). (*f*) However, there was an increase in subsequent offspring fecundity with increased maternal fecundity for women from landowning families (slope estimate 0.31±0.040). The raw data points appear in the background. Vertical dashed lines show the population mean maternal fecundity (6.61). Horizontal dashed lines show the population geometric means for (*a*,*b*) grand-offspring number per birth (1.93), (*c*,*d*) the proportion of offspring recruited (0.45) and (*e*,*f*) average subsequent offspring fecundity (4.29). Parameter estimates are derived from analyses presented in table 4.

**Table 1:** Women from landowning families have more offspring, recruited offspring (survived to breed) and grand-offspring compared with women from landless families. (There were 248 landless women and 189 women from landowning families. The sample size (*n*) of the numbers of offspring, recruited offspring and grand-offspring is shown for each socio-economic category. We applied Box–Cox power transformations to each of the three response variables and investigated the influence of socio-economic status using linear models.)

	*n*	mean±s.e.	d.f.	*F*	*p*
*offspring*					
socio-economic status			1,435	41.52	<0.01
landless	1464	5.90±0.17			
versus landowning	1424	7.53±0.19			
*recruited offspring*					
socio-economic status			1,435	28.26	<0.01
landless	659	2.66±0.11			
versus landowning	672	3.56±0.13			
*grand-offspring*					
socio-economic status			1,435	34.7	<0.01
landless	3039	12.25±0.70			
versus landowning	3431	18.15±0.85			

**Table 2:** Socio-economic status significantly changed the way maternal fecundity and the proportion of offspring recruited combine to predict grand-offspring number. (This was shown by the reduction in model AIC when socio-economic status was added as a two-level factor, and this was further confirmed with a likelihood-ratio test. The likelihood-ratio tests presented compare each model with that in the row above it. The model is a GLMM with a Poisson error structure and a log link function that had random intercepts for each parish and 10-year maternal birth cohort. d.f.^1^ are the total degrees of freedom taken up by the terms in the model. d.f.^2^ are the degrees of freedom for the *Χ*^2^ statistic in the likelihood-ratio test. The minimal adequate model (MAM) was obtained by the removal of the three-way and 2 two-way interaction terms. ^*^ indicates the terms deleted to reach the MAM. Eligible terms were removed one at a time and their contribution to model explanatory power assessed using likelihood-ratio tests and by comparing the AIC of models with and without each term. The term that caused the biggest reduction in model AIC was deleted and the process repeated until the MAM was reached.)

	d.f.^1^	AIC	d.f.^2^	*Χ*^2^	*p*
maximal model without socio-economic status	6	919.57			
maximal model with socio-economic status	12	890.88	6	40.69	<0.01
*minus* fecundity×recruitment×socio-economic status^*^	11	889.08	1	0.21	0.65
*minus* recruitment^2^×socio-economic status^*^	10	888.37	1	1.29	0.26
*minus* fecundity×recruitment^*^	9	887.83	1	1.46	0.23

**Table 3:** Parameter estimates from the MAM of the effects of maternal fecundity and the proportion of offspring recruited on grand-offspring number in landowning and landless families. (The relative differences in the parameter estimates for the landowning compared with the landless socio-economic group are given in the row below the parameter estimates for the landless socio-economic group. The model is a GLMM with a Poisson error structure and a log link function that had random intercepts for each parish and 10-year maternal birth cohort. The Poisson parameter estimates were obtained using a Laplace approximation to maximum likelihood. 95% CIs are the Bayesian HPD 95% CIs of the MCMC sample for each parameter. The *p*-values based on the normal (*z*) distribution show the same pattern as the 95% CIs, which are based on the underlying Poisson distribution of the model residuals.)

	estimate	95% CI	*z*	*p*
landless (intercept)	−1.20	−1.63 to −0.78	−7.06	<0.01
versus landowning	0.97	0.59 to 1.38	4.88	<0.01
fecundity (landless)	0.37	0.31 to 0.44	11.79	<0.01
versus landowning	−0.18	−0.27 to −0.10	−4.25	<0.01
fecundity^2^ (landless)	−0.014	−0.018 to −0.010	−6.90	<0.01
versus landowning	0.012	0.0065 to 0.017	4.48	<0.01
recruitment (landless)	5.37	4.76 to 5.98	17.31	<0.01
versus landowning	−0.53	−0.82 to −0.26	−3.74	<0.01
recruitment^2^	−2.57	−3.08 to −2.11	−10.39	<0.01

**Table 4:** The linear model parameter estimates for the effects of maternal fecundity on the geometric means of three measures of offspring quality: (i) grand-offspring per birth (overall offspring quality), (ii) the proportion of offspring recruited, and (iii) average subsequent fecundity of recruited offspring.

	d.f.	*F*	*p*
(i) *overall offspring quality*			
fecundity	1,26	2.78	0.11
socio-economic status	1,26	2.27	0.14
fecundity×socio-economic status	1,26	4.23	0.05
(ii) *offspring recruitment*			
fecundity	1,26	33.66	<0.01
socio-economic status	1,26	0.68	0.42
fecundity×socio-economic status	1,26	1.59	0.22
(iii) *subsequent offspring fecundity*			
fecundity	1,26	69.27	<0.01
socio-economic status	1,26	17.39	<0.01
fecundity×socio-economic status	1,26	25.25	<0.01
